# Molecular docking and molecular dynamics study Lianhua Qingwen granules (LHQW) treats COVID-19 by inhibiting inflammatory response and regulating cell survival

**DOI:** 10.3389/fcimb.2022.1044770

**Published:** 2022-11-24

**Authors:** Jun-Feng Cao, Yunli Gong, Mei Wu, Li Xiong, Shengyan Chen, Haonan Huang, Xinge Zhou, Ying-chun Peng, Xue-fang Shen, Jinyu Qu, Yi-li Wang, Xiao Zhang

**Affiliations:** ^1^ Chengdu Medical College, Chengdu, China; ^2^ Chengdu Medical College of Basic Medical Sciences, Chengdu, China; ^3^ The First Affifiliated Hospital of Chengdu Medical College, Chengdu, China

**Keywords:** COVID-19, lianhua qingwen granules (LHQW), molecular docking, bioinformatics analysis, molecular dynamics

## Abstract

**Purpose:**

2019 Coronavirus disease (COVID-19) is endangering health of populations worldwide. Latest research has proved that *Lianhua Qingwen granules* (LHQW) can reduce tissue damage caused by inflammatory reactions and relieve patients’ clinical symptoms. However, the mechanism of LHQW treats COVID-19 is currently lacking. Therefore, we employed computer simulations to investigate the mechanism of LHQW treats COVID-19 by modulating inflammatory response.

**Methods:**

We employed bioinformatics to screen active ingredients in LHQW and intersection gene targets. PPI, GO and KEGG was used to analyze relationship of intersection gene targets. Molecular dynamics simulations validated the binding stability of active ingredients and target proteins. Binding free energy, radius of gyration and the solvent accessible surface area were analyzed by supercomputer platform.

**Results:**

COVID-19 had 4628 gene targets, LHQW had 1409 gene targets, intersection gene targets were 415. Bioinformatics analysis showed that intersection targets were closely related to inflammation and immunomodulatory. Molecular docking suggested that active ingredients (including: licopyranocoumarin, Glycyrol and 3-3-Oxopropanoic acid) in LHQW played a role in treating COVID-19 by acting on CSF2, CXCL8, CCR5, NLRP3, IFNG and TNF. Molecular dynamics was used to prove the binding stability of active ingredients and protein targets.

**Conclusion:**

The mechanism of active ingredients in LHQW treats COVID-19 was investigated by computer simulations. We found that active ingredients in LHQW not only reduce cell damage and tissue destruction by inhibiting the inflammatory response through CSF2, CXCL8, CCR5 and IFNG, but also regulate cell survival and growth through NLRP3 and TNF thereby reducing apoptosis.

## Graphical Abstract

The mechanisms analysis of *Lianhua Qingwen granules* (LHQW) treats COVID-19.

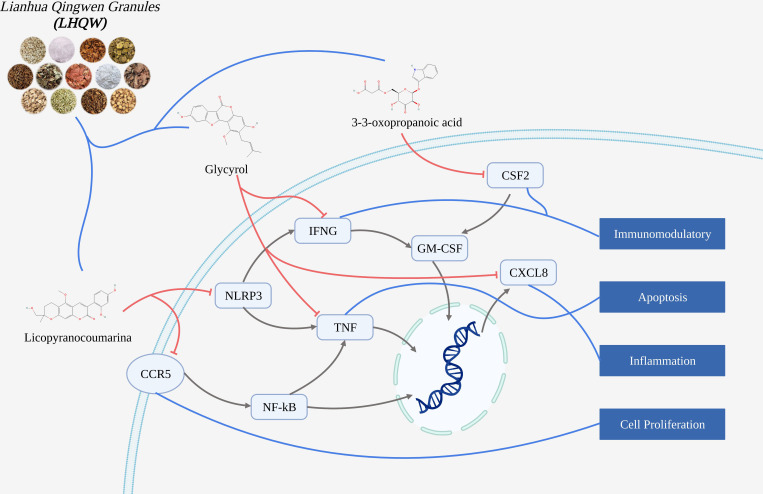

## Introduction

SARS-CoV-2 is a single-stranded RNA virus that belongs to the beta family of coronaviruses (Mahmoudinobar et al., 2021; Bui et al., 2021). The virus has a spherical lipid bilayer envelope with a spike (S) protein on its surface responsible for viral infection (Ilkhani et al., 2021; Qasem et al., 2021). The genome of SARS-CoV-2 is engaged by two open reading frames, they encode Nuclear Protein Phosphatase 1 α (PP1A) and Replicase polyprotein 1ab (pp1ab) (Citarella et al., 2021). The coronavirus protease M^pro^ (also known as 3C-like protease (3CL^pro^)) is a triple structural domain cysteine protease and M^pro^ is involved in the proteolytic processing of pp1a and pp1ab (Panikar et al., 2021). Numerous studies have suggested a critical role for the protease M^pro^ in coronavirus gene expression and replicase processing (Roe et al., 2021), and M^pro^ mediates viral replication and transcription (Sabbah et al., 2021). Study has been suggested that targeting and inhibiting M^pro^ activity is an effective way to prevent SARS-CoV-2 replication and transmission (Sultan et al., 2021). DasGupta et al., 2022 used mixed solvent molecular dynamics (MixMD) to simulate and analyze the possible metastable sites of M^pro^. Sztain et al., 2020 identified cryptic pocket structures within M^pro^ of SARS-CoV-2 by Gaussian accelerated molecular dynamics, and these pocket structures may serve as drug targets to develop protease inhibitors of COVID-19. Strömich et al., 2022 found by computer simulation that M^pro^ has multiple potentially acting binding sites capable of interfering with viral RNA transcription and protein translation. Günther et al., 2021 identified two metastable binding sites of M^pro^ as possible drug targets against SARS-CoV-2 by high-throughput X-ray crystallography analysis.

The clinical presentation of patients with COVID-19 ranges from mild flu-like symptoms to life-threatening multi-organ failure. (Sidiq et al., 2020). Studies have found that critically ill patients may develop acute myocardial injury, renal dysfunction and coagulation complications (Rello et al., 2020; Shi et al., 2021; Ciotti et al., 2021). Although relevant vaccines and drugs have been used clinically, clinical studies have found many adverse events (such as: fever, headache, muscle pain and joint pain) after vaccination in some populations (Vitiello et al., 2021; Li Z et al., 2020). The Ad26.COV2.S vaccine produced by Johnson had 34% untreated rate of vaccine efficacy against COVID-19 in a phase 3 clinical trial. (Hadj Hassine, 2022). Gam-COVID-Vac (Sputnik V) is a heterologous adenovirus vector vaccine based on SARS-CoV-2, its phase 3 clinical trial found mild adverse reactions in 94% of subjects, with a very small number of subjects experiencing serious adverse events. And there were four vaccine-related deaths in subjects (Fernandes et al., 2022). Both the Pfizer and Modena COVID-19 vaccines granted by the U.S. Food and Drug Administration (FDA) have shown some associated adverse reactions (Meo et al., 2021; Ng et al., 2021; Yamamoto, 2022). The antiviral drug raltegravir is the only drug approved for the treatment of COVID-19. Although there is little information on the adverse events of raltegravir, studies have reported that raltegravir use is associated with an increase in renal disease (Martinez-Lopez et al., 2020; Chouchana et al., 2021). Famipiravir is one of the anti-SARS-CoV-2 candidates, clinical trials have found adverse effects such as vomiting chest pain and elevated serum liver transaminase and uric acid levels in mild to moderate patients (Hassanipour et al., 2021).

In the treatment for COVID-19, China has always adhered to the principle of “treating both Chinese and Western medicine”, and the Chinese herbal medicine *Lianhua Qingwen granules* (LHQW) has played an important role in the fight against the epidemic (Zeng et al., 2020). LHQW is a herbal formula consisting of 13 herbs, including forsythia, jinyinhua, ephedra, almonds, gypsum, panax quinquefolium, cotton horse kanji, fishy grass, patchouli, rhubarb, licorice, rhodiola, and menthol. LHQW has broad-spectrum antiviral, effective antibacterial, antipyretic and anti-inflammatory, anti-cough and cough, and immunomodulatory functions (Zhang X et al., 2020). Studies have shown that LHQW has good effects on various influenza virus-induced respiratory diseases (Zeng et al., 2020). Clinical studies have shown that Lotus Clear can improve clinical symptoms (such as: fever, fatigue and muscle aches) and shorten the duration of COVID-19 in patients with COVID-19. Experimental studies have also shown that lotus seeds can exert pharmacological effects in the treatment of COVID-19 by inhibiting viral replication and reducing cytokine release from host cells (Liu C et al., 2021; Runfeng et al., 2020; Hu et al., 2021). LHQW is commonly used as an adjuvant to COVID-19, and it is thought to provide symptomatic relief and shorten the course of the disease (Wang et al., 2020). Study found that LHQW reduced D-dimer (prognostic indicator of pneumonia) and erythrocyte sedimentation rate (marker of inflammation) in patients, thereby delaying disease progression (Shen et al., 2021). LHQW was found to improve not only the clinical symptoms but also the prognosis of patients with COVID-19 (Xiao et al., 2020; Li L. et al., 2020; Zeng et al., 2020; Zhuang et al., 2021). A pharmacodynamic study from 284 patients with neocrown pneumonia in China showed a cure rate of 91.5% in the LHQW treatment group, which was significantly higher than the 82.4% cure rate in the conventional group (Wu and Zhong, 2021). And clinical studies have shown that LHQW has improved the symptoms of fever, fatigue and cough in COVID-19 patients (Tong et al., 2020; Wang et al., 2022). Although LHQW has played a positive role in the treatment of COVID-19, the complex composition has hindered a more in-depth study of the pharmacological mechanism of action of lotus clematis in the treatment of COVID-19.

Therefore, we further investigated the mechanism of LHQW treats COVID-19 by bioinformatics analysis and computer simulation. Molecular docking and molecular dynamics can allow for a comprehensive simulation of the interaction and binding stability among ligand active ingredients and receptor protein targets with the help of powerful computational capabilities.

In this study, bioinformatics was used to obtain active ingredients in LHQW and intersection gene targets. computer simulations were validated the relationship among active ingredients and protein targets by supercomputer platform. Molecular docking was used to validate affinity of ligand active ingredients and receptor proteins. Molecular dynamics was used to simulate the stability of binding complex.

## Material and methods

### Screening active ingredients and gene targets

Traditional Chinese Medicine Systems Pharmacology Database (TCMSP) was used to screen and analyse ingredients in *Lianhua Qingwen granules* (LHQW) (Mok et al., 2022). And active ingredients in LHQW were screened by the criterion of OB≥80% and DL≥0.2. Oral bioavailability (OB) is defined as the extent to which the active ingredient is utilized by the body, and OB can determine the impact of a compound on disease (Zhang Y. et al., 2020). In drug development, drug likeness (DL) is important to improve the success of drug discovery and development (Wu et al., 2022). GeneCards database was used to obtain disease gene targets and drug gene targets (Stelzer et al., 2016). Disease gene targets and drug gene targets were combined by Venny website to obtain intersection gene targets.

### Protein-protein interaction network and enrichment analysis

The STRING database was employed to analyze protein-protein interaction (PPI) of *Lianhua Qingwen granules* (LHQW) treats COVID-19 (Szklarczyk et al., 2021). And core interaction protein targets were screened according to the criteria of nodal degree value and median centroid value greater than the mean. DAVID database was employed for gene ontology (GO) and Kyoto Encyclopedia of Genes and Genomes (KEGG) enrichment analysis (Wu et al., 2020). This study obtained cellular component (CC), molecular function (MF) and biological process (BP) of the gene targets through GO enrichment. KEGG pathway enrichment analysis was performed on the relevant signaling pathways involved in interaction gene targets (Cao et al., 2022a).

### Molecular docking

The Pubchem database was used to obtain 3D structures of small molecules, and the PDB database was used to obtain the structures of proteins (Jagdish and Marty Ytreberg, 2019; Zhou et al., 2020; Adegbola et al., 2021; Dela Cruz et al., 2022). Small molecules and proteins were subjected to energy minimization under MMFF94 force field. AutoDock Vina 1.1.2 software was used for molecular docking (Apriyanti et al., 2021). All protein seat receptors were pre-treated (including the removal of water molecules, salt ions, and other small molecules from the protein results) by PyMol 2.5 (Kashyap et al., 2021). The center of the docking box was defined using PyMol based on the location of the active site and the box side length was set to 22.5 Å. ADFRsuite 1.0 was used to convert all processed small molecules and receptor proteins into the PDBQT format required for molecular docking (Song et al., 2019; Kumar et al., 2020). The highest scoring docked conformations from the molecular docking output were used for subsequent molecular dynamics simulations (Cao et al., 2022b). We used the original crystal ligand of the target protein as a positive reference, and analyzed and compared the binding posture of the original crystal ligand and protein, the chemical bond length and the chemical bond angle by re-docking the original crystal ligand and protein. Finally, the consistency of the binding mode can indicate the correctness of the molecular docking protocol.

### Molecule dynamics

The highest scoring conformations identified by molecular docking analysis were further validated by molecular dynamics simulation. Molecular dynamics (MD) simulation is based on Newtonian mechanics theory to model the motion of molecular systems (Jagdish et al., 2014). AMBER 18 software was used to perform molecular dynamics simulations (Mermelstein et al., 2018; Lee et al., 2018). GAFF2 small molecule force fields and ff14SB protein force fields were used to treat ligand small molecules and receptor proteins (Lee J. et al., 2020). Hydrogen atoms were added to all complex systems using the LEaP module and a truncated octahedral TIP3P solvent cassette was added at a distance of 10 Å from the system. Na+/Cl- was added to balance the charge of the complex system (Rieloff and Skepö, 2021). The steepest descent method with 2500 steps and the conjugate gradient method with 2500 steps were used for the energy optimization of the complex system (Lee, T. S. et al., 2020). After the energy optimization of the system was completed, the system temperature was steadily increased from 0 K to 298.15 K at a fixed volume and constant ramp rate. The NVT (Isothermal isovolume) system simulation of 500 ps was performed to further uniformly distribute the solvent molecules in the solvent box at the system maintenance temperature of 298.15 K (Cao et al., 2022c). Finally, the composite system was subjected to a 100 ns NPT (isothermal isobaric) system simulation. The truncation distance of the non-bond was set to 10 Å during the molecular dynamics simulation. Particle mesh Ewald (PME) was used to calculate electrostatic interactions. SHAKE method was used to limit the bond length of hydrogen atoms. Langevin algorithm was used for temperature control of the simulation, and the collision frequency was set to 2 ps^-1^. The simulated system pressure was 1 atm, the integration step was 2 fs, and the traces were saved at 10 ps intervals for subsequent analysis. The binding free energy can reflect the interaction between the ligand small molecule and the receptor protein (Wang et al., 2021). The interactions between ligand and receptor include both covalent and non-bonded interactions, but only non-bonded interactions (van der Waals interactions, electrostatic interactions and hydrogen bonding interactions) are generally present in complex systems of drug small molecules (ligands) and proteins (receptors) (Dasmahapatra et al., 2022). Therefore, AMBER 18 was used to calculate the binding free energy by MM/GBSA method in this study (Weng et al., 2019; Cao et al., 2022d).

## Results

### Active ingredientsand intersection gene targets

**Figure 1:**
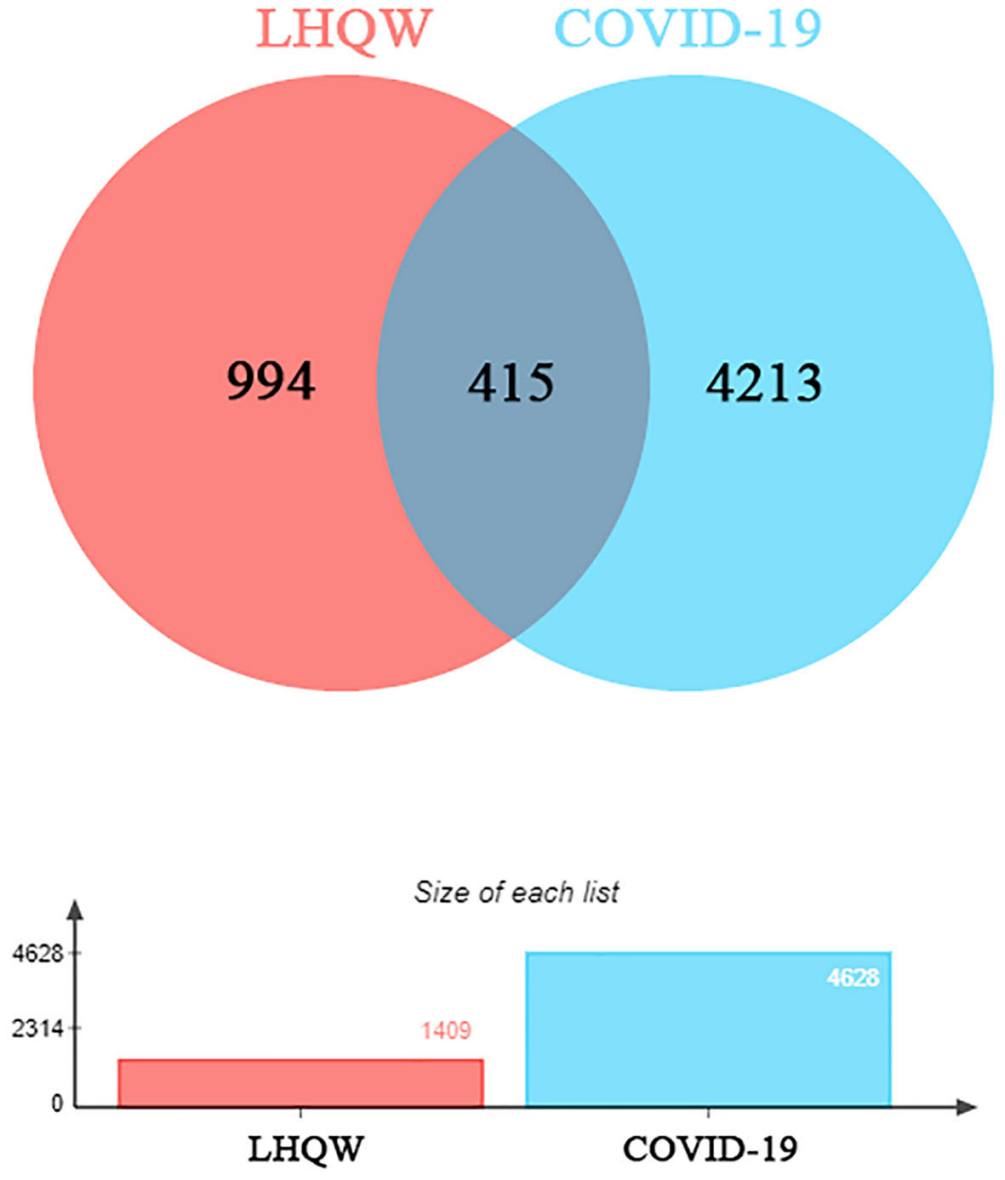
The venny of intersection gene targets. Targets of the intersection of *Lianhua Qingwen granules* (LHQW) and COVID-19.

1391 ingredients in *Lianhua Qingwen granules* (LHQW) were obtained from TCMSP database. 10 active ingredients were screened by using DL≥0.2 and OB≥80% as the criteria (**Table 1**). GeneCards database was used to obtain 4628 COVID-19 gene targets and 1409 *Lianhua Qingwen granules* (LHQW) gene targets. And 415 intersection gene targets were processed by Venny (**Figure 1**).

**Table 1:** The active ingredients in Lianhua Qingwen granules (LHQW).

The active ingredients in Lianhua Qingwen granules (LHQW)							
Mol ID	Molecule Name	MW	AlogP	OB (%)	BBB	DL	HL
**MOL003330**	Phillygenin	372.45	2.38	95.04	0.07	0.57	1.97
**MOL002311**	Glycyrol	366.39	4.85	90.78	-0.2	0.67	9.85
**MOL003006**	(3R,8S,9R,9aS,10aS)-9-ethenyl-8-(beta-D-glucopyranosyloxy)-2,3,9,9a,10,10a-hexahydro-5-oxo-5H,8H-pyrano-pyridine-3-carboxylic acid	281.29	-0.96	87.47	-0.89	0.23	5.5
**MOL012922**	I-SPD	327.41	3.1	87.35	0.21	0.54	1.68
**MOL001734**	3-3-oxopropanoic acid	379.35	0.36	85.87	-1.25	0.47	3.58
**MOL003306**	Pinoresinol monomethyl ether	372.45	2.38	85.12	0	0.57	2.12
**MOL004990**	7,2’,4’-trihydroxy-5-methoxy-3-arylcoumarin	300.28	2.56	83.71	-0.59	0.27	0.99
**MOL000471**	Aloe-emodin	270.25	1.67	83.38	-1.07	0.24	31.49
**MOL003322**	Forsythinol	372.45	2.38	81.25	-0.08	0.57	2.72
**MOL004904**	Licopyranocoumarin	384.41	3.04	80.36	-0.62	0.65	0.08

MW, molecular weight.

AlogP, partition coefficient between octanol and water.

OB, oral bioavailability.

BBB, blood brain barrier.

DL, drug similarity.

HL, drug half-life.

### Core intersection target screening and Protein-protein interaction network constructing

**Figure 2:**
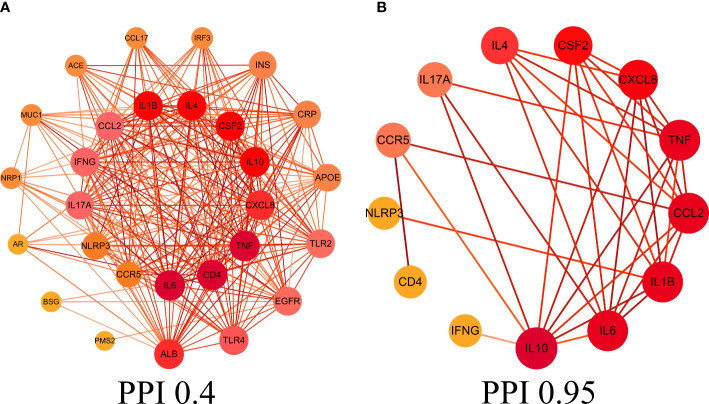
Protein-protein interaction (PPI) network. **(A)** PPI network of protein targets, **(B)** PPI network of core protein targets (confidence>0.95).

This study obtained 29 core intersection targets by relevance score (relevance score≥4). STRING database was used to analysis core intersection targets of LHQW and COVID-19, 13 core protein targets (including: NLRP3, TNF, CSF2, IFNG) were obtained by setting confidence degree (confidence degree>0.95) (**Figures 2A, B**).

### GO and KEGG enrichment analysis

**Figure 3:**
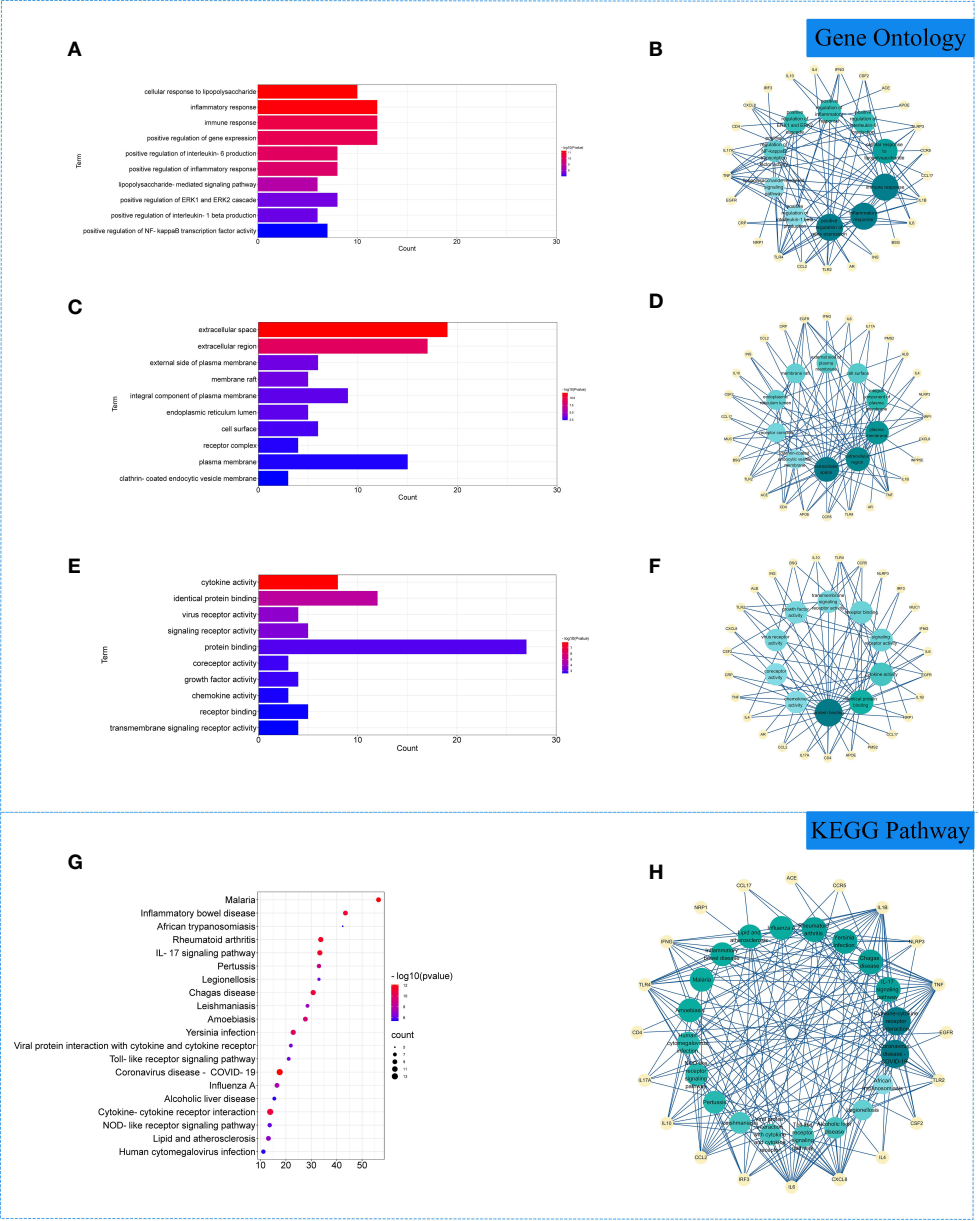
Gene Ontology (GO) and Kyoto Encyclopedia of Genes and Genomes (KEGG) analysis of related genes. **(A)** The top 10 terms in biological processes (BP) were greatly enriched. **(B)** The subnetwork displayed the top 10 BP terms and related genes. **(C)** The top 10 terms in cellular components (CC) were greatly enriched. **(D)** The subnetwork displayed the first 10 CC terms and related genes. **(E)** The top 10 terms in molecular function (MF) were greatly enriched. **(F)** The subnetwork displayed the top 10 MF terms and related genes. **(G)** The top 20 KEGG pathways were showed. **(H)** The subnetworks displayed the top 20 KEGG pathways and related.

The 29 intersection gene targets were imported into DAVID database for GO and KEGG enrichment analysis. GO enrichment analysis yielded a total of 267 GO, including 224 biological processes (BP), 19 cell components (CC) and 24 molecular functions (MF). Biological processes were correlated with cellular response to lipopolysaccharide and inflammatory response. Among cell components, extracellular space and integral component of plasma membrane for a relatively large amount. In molecular functions, virus receptor activity and virus receptor activity were relatively high (**Figures 3A–F**). KEGG pathway yielded 71 pathways, and enriched pathways involved rheumatoid arthritis, coronavirus disease - COVID-19 and other signaling pathways (**Figures 3G, H**).

### Molecular docking

**Figure 4:**
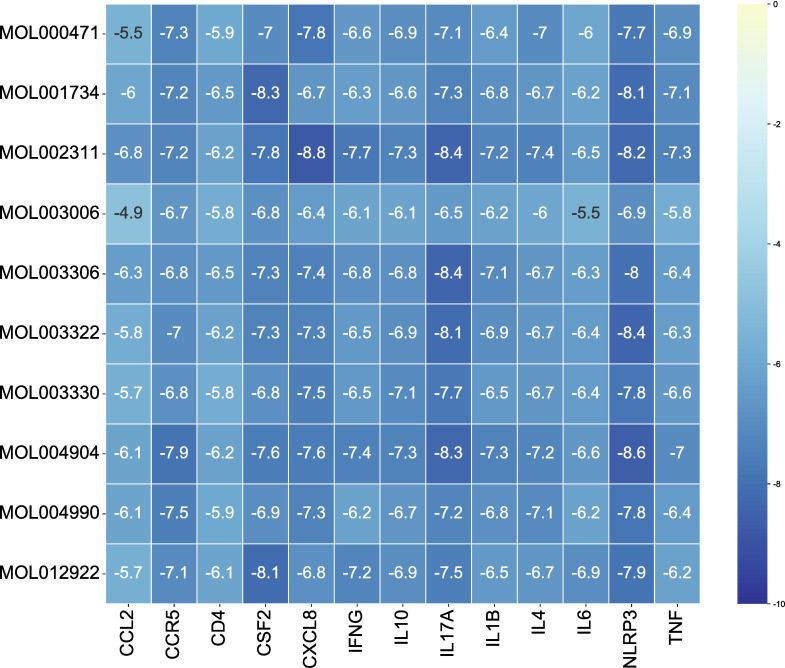
The result of molecular docking.

**Figure 5:**
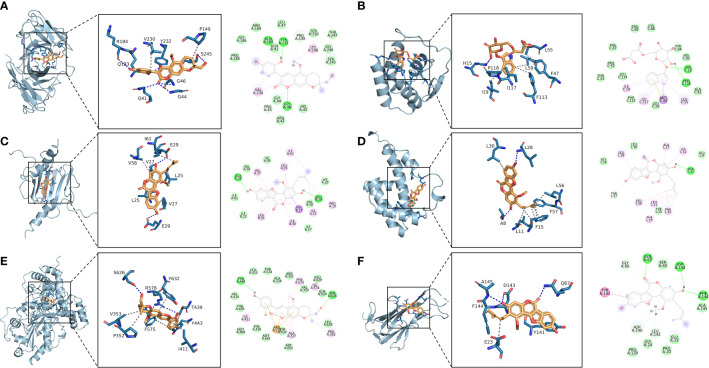
Binding conformation of active ingredients and protein targets. **(A)** CCR5/Licopyranocoumarin, **(B)** CSF2/3-3-Oxopropanoic acid, **(C)** CXCL8/Glycyrol, **(D)** IFNG/Glycyrol, **(E)** NLRP3/Licopyranocoumarin, **(F)** TNF/Glycyrol.

The 10 active ingredients and 13 core intersection protein targets were used for molecular docking. The stability of protein receptor-small molecule ligand binding depends on the binding energy. The lower the binding energy of the complex, the more stable the receptor-ligand binding conformation (**Figure 4**). Molecular docking indicated that CCR5/Licopyranocoumarin was mainly maintained by hydrogen bonding and hydrophobic interactions. Licopyranocoumarin interacted with Arg-184, Tyr-232, Gln-41, Gly-44, Gly-46 and Ser-245 on the CCR5 protein by hydrogen bonding and with Val-230 and Pro-149 by hydrophobic interactions (**Figure 5A**). The binding of CSF2/3-3-Oxopropanoic acid was maintained mainly by hydrophobic interactions. 3-3-Oxopropanoic acid interacted with His-15 on the CSF2 protein by hydrogen bonding and with Leu-55, Leu-59, Phe-47, Ile-117 and Ile-19 by hydrophobic interactions (**Figure 5B**). In the CXCL8/Glycyrol, Glycyrol interacted with Glu-29 and Val-27 on CXCL8 by hydrogen bonding and with Val-58, Ile-61, Leu-25 and Val-27 by hydrophobic interactions (**Figure 5C**). The binding of IFNG/Glycyrol indicated that Glycyrol hydrogen bonds with Ala-8 and Leu-28 on the protein, hydrophobic interaction with Leu-11, Phe-15, Phe-57, Leu-57 and Leu-30 (**Figure 5D**). Licopyranocoumarin could form hydrogen bonding with Ser-626 and Arg-578 on NLRP3 protein, and also with Val-353, Pro-352, Tyr-632, Ile-411, Phe-575 and Thr-439 formed a hydrophobic interaction (**Figure 5E**). The binding of TNF/Glycyrol was mainly through hydrophobic interaction, Glycyrol interacted with Phe-144, Ala-145, Asp-143, Gln-67 and Tyr-141 on TNF protein by hydrophobic interactions and with Glu-23 by hydrogen bonding (**Figure 5F**).

### Molecular dynamics and binding free energy

**Figure 6:**
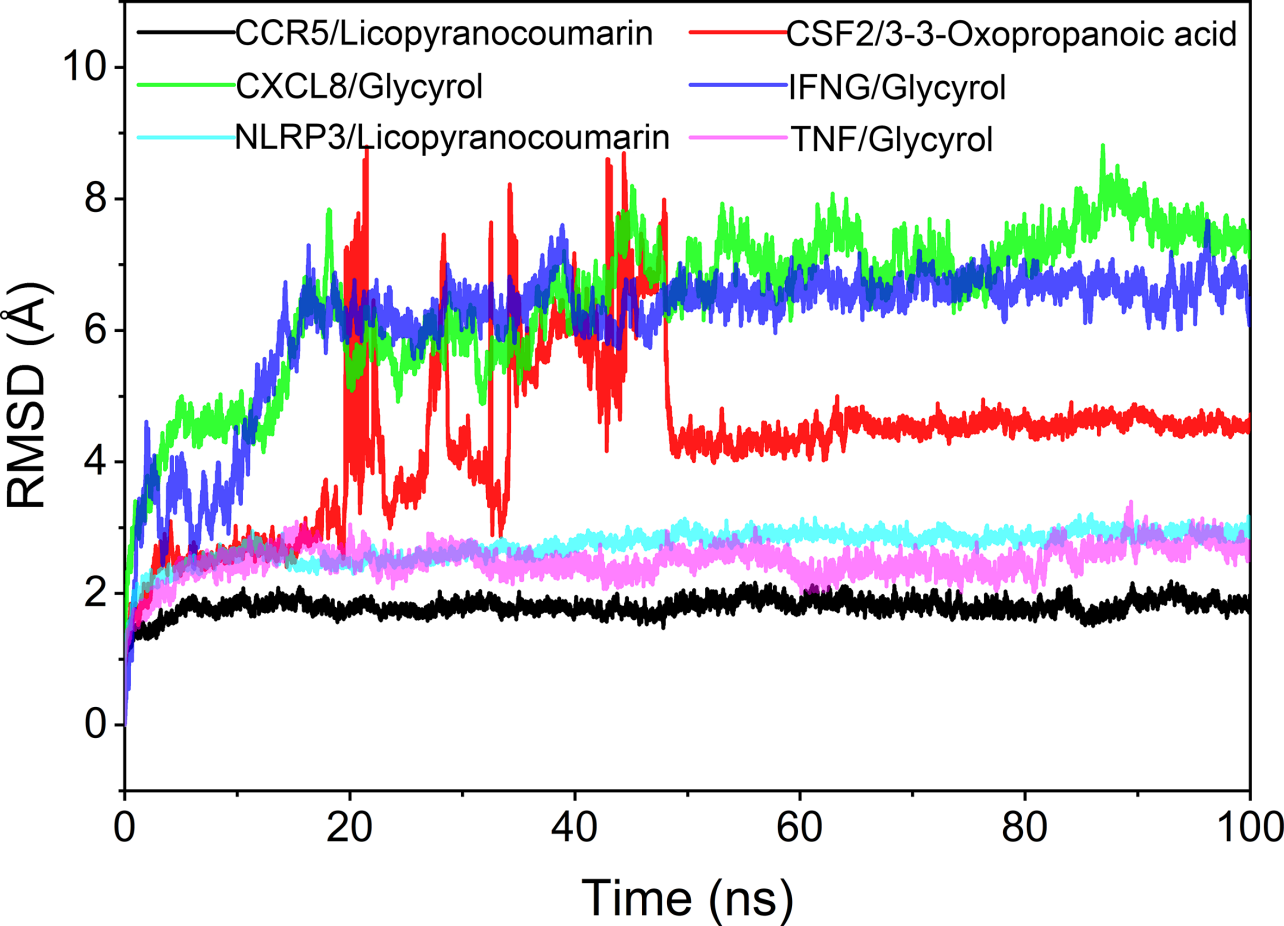
The result of root mean square deviation (RMSD).

The root mean square deviation (RMSD) was used to reflect the fluctuating processes of the complexes. Higher RMSD values and fluctuations of the complexes indicate more intense motions of protein receptors and small molecule ligands. The MMGBSA method was used to calculate the binding free energy of the complexes, and the binding free energy can more accurately reflect the magnitude of the binding stability of the ligand small molecule to the receptor protein (Procacci, 2020). The binding free energy reflects the energy released by the binding of the ligand and receptor into the complex (Al-Khafaji and Taskin Tok, 2020). The higher the value of the binding free energy, the more stable the structure of the complex. Except for CSF2/3-3-Oxopropanoic acid, the remaining five complexes gradually converged within the first 5 ns of the simulation and remained stable in the subsequent simulations. In particular, RMSD values of three complexes (CCR5/Licopyranocoumarin, NLRP3/Licopyranocoumarin and TNF/Glycyrol) were small (values below 3.5 Å) and fluctuated very stably. CSF2/3-3-Oxopropanoic acid and IFNG/Glycyrol both showed significant fluctuations in the first 50 ns, but they rapidly stabilized after 50 ns. CXCL8/Glycyrol was never in a stable fluctuation throughout the simulation, indicating the relatively poor stability of CXCL8/Glycyrol (**Figure 6**). The binding free energies of CCR5/Licopyranocoumarin, CSF2/3-3-Oxopropanoic acid, CXCL8/Glycyrol, IFNG/Glycyrol, NLRP3/Licopyranocoumarin and TNF/Glycyrol were -25.52 ± 2.86 kcal/mol, -6.98 ± 5.02 kcal/mol, -39.15 ± 1.96 kcal/mol, -34.16 ± 4.59 kcal/mol, -38.17 ± 1.51 kcal/mol and -16.84 ± 1.92 kcal/mol (**Table 2**). The stability of CCR5/Licopyranocoumarin, CXCL8/Glycyrol and IFNG/Glycyrol was good and their values of binding free energy were greater than -30.0 kcal/mol. We found that the binding free energies of all complexes were mainly Van der Waals and electrostatic energies.

**Table 2:** Binding free energies and energy components predicted by MM/GBSA (kcal/mol).

Results of binding free energies						
System name	CCR5/Licopyranocoumarin	CSF2/3-3-Oxopropanoic acid	CXCL8/Glycyrol	IFNG/Glycyrol	NLRP3/Licopyranocoumarin	TNF/Glycyrol
**Δ*E*_vdw_**	-31.41 ± 2.76	-11.53 ± 6.29	-46.22 ± 1.20	-41.36 ± 1.57	-43.33 ± 2.46	-26.13 ± 2.16
**Δ*E*_elec_**	-25.44 ± 5.08	-12.81 ± 38.54	-8.04 ± 2.37	-18.88 ± 9.99	-35.40 ± 2.72	-13.98 ± 5.68
**ΔG_GB_**	5.62 ± 3.49	19.08 ± 40.98	20.90 ± 1.70	31.88 ± 5.76	46.01 ± 3.84	25.71 ± 5.40
**ΔG_SA_**	-4.29 ± 0.24	-1.72 ± 1.01	-5.78 ± 0.16	-5.80 ± 0.17	-6.44 ± 0.10	-2.44 ± 0.31
**ΔG_bind_**	-25.52 ± 2.86	-6.98 ± 5.02	-39.15 ± 1.96	-34.16 ± 4.59	-38.17 ± 1.51	-16.84 ± 1.92

ΔE_vdW_, van der Waals energy.

ΔE_elec_, electrostatic energy.

ΔG_GB_, electrostatic contribution to solvation.

ΔG_SA_, non-polar contribution to solvation.

ΔG_bind_, binding free energy.

### Analysis of root mean square fluctuations

**Figure 7:**
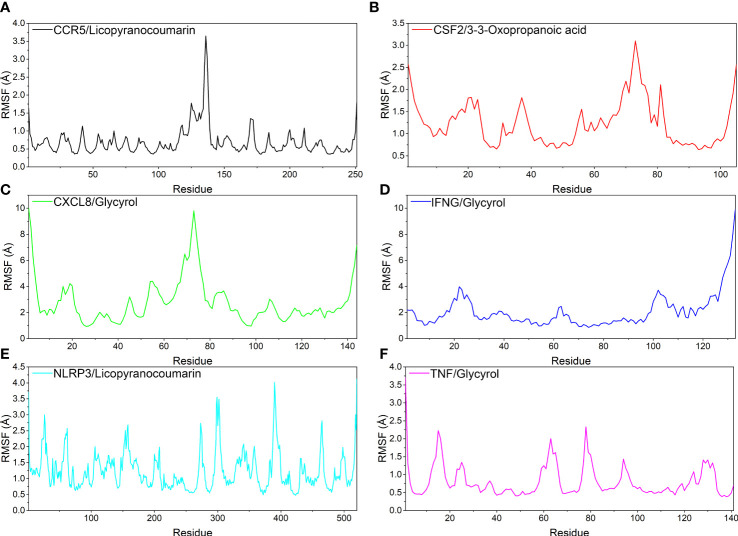
The result of root mean square fluctuations (RMSF). **(A)** CCR5/Licopyranocoumarin, **(B)** CSF2/3-3-Oxopropanoic acid, **(C)** CXCL8/Glycyrol, **(D)** IFNG/Glycyrol, **(E)** NLRP3/Licopyranocoumarin, **(F)** TNF/Glycyrol.

In this study, root mean square fluctuations (RMSF) were used to model the vibrations of each residue after ligand and receptor binding to explore the fluctuating changes of macromolecular proteins at the residue level. Typically, the flexibility of a protein decreases upon binding of a ligand small molecule drug to a receptor protein, thereby modulating the active site of the protein. The simulation results show that all proteins except the ends of the protein have low RMSF after binding different small molecules, indicating that the core structure of the protein possesses good rigidity. Notably, the overall RMSFs of CCR5/Licopyranocoumarin, CSF2/3-3-Oxopropanoic acid, IFNG/Glycyrol, NLRP3/Licopyranocoumarin and TNF/Glycyrol were all less than 2.5 Å, indicating that the rigidity of the proteins increased significantly after the binding of proteins to small molecules (**Figure 7**).

### Analysis of hydrogen bond

**Figure 8:**
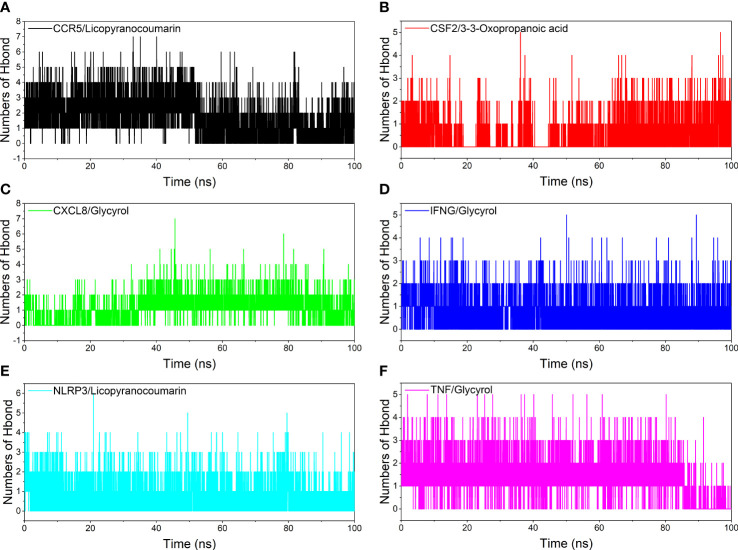
Analysis of hydrogen bonds. **(A)** CCR5/Licopyranocoumarin, **(B)** CSF2/3-3-Oxopropanoic acid, **(C)** CXCL8/Glycyrol, **(D)** IFNG/Glycyrol, **(E)** NLRP3/Licopyranocoumarin, **(F)** TNF/Glycyrol.

Hydrogen bonding is one of the strongest non-covalent binding interactions between receptor and ligand, and the higher the number of hydrogen bonds, the better the binding stability. CCR5/Licopyranocoumarin with a stable number of hydrogen bonds of about 3 throughout; followed by NLRP3/Licopyranocoumarin and CXCL8/Glycyrol with the number of hydrogen bonds remaining at 2-3 at the late stage of the simulation. We found that CSF2/3-3-Oxopropanoic acid and CXCL8/Glycyrol showed a significant decrease in hydrogen bonding in the early stage of the simulation, and this result further explains the anomalous fluctuations of CSF2/3-3-Oxopropanoic acid and CXCL8/Glycyrol in the early stage of RSMD (**Figure 8**).

### Analysis of the radius of gyration

**Figure 9:**
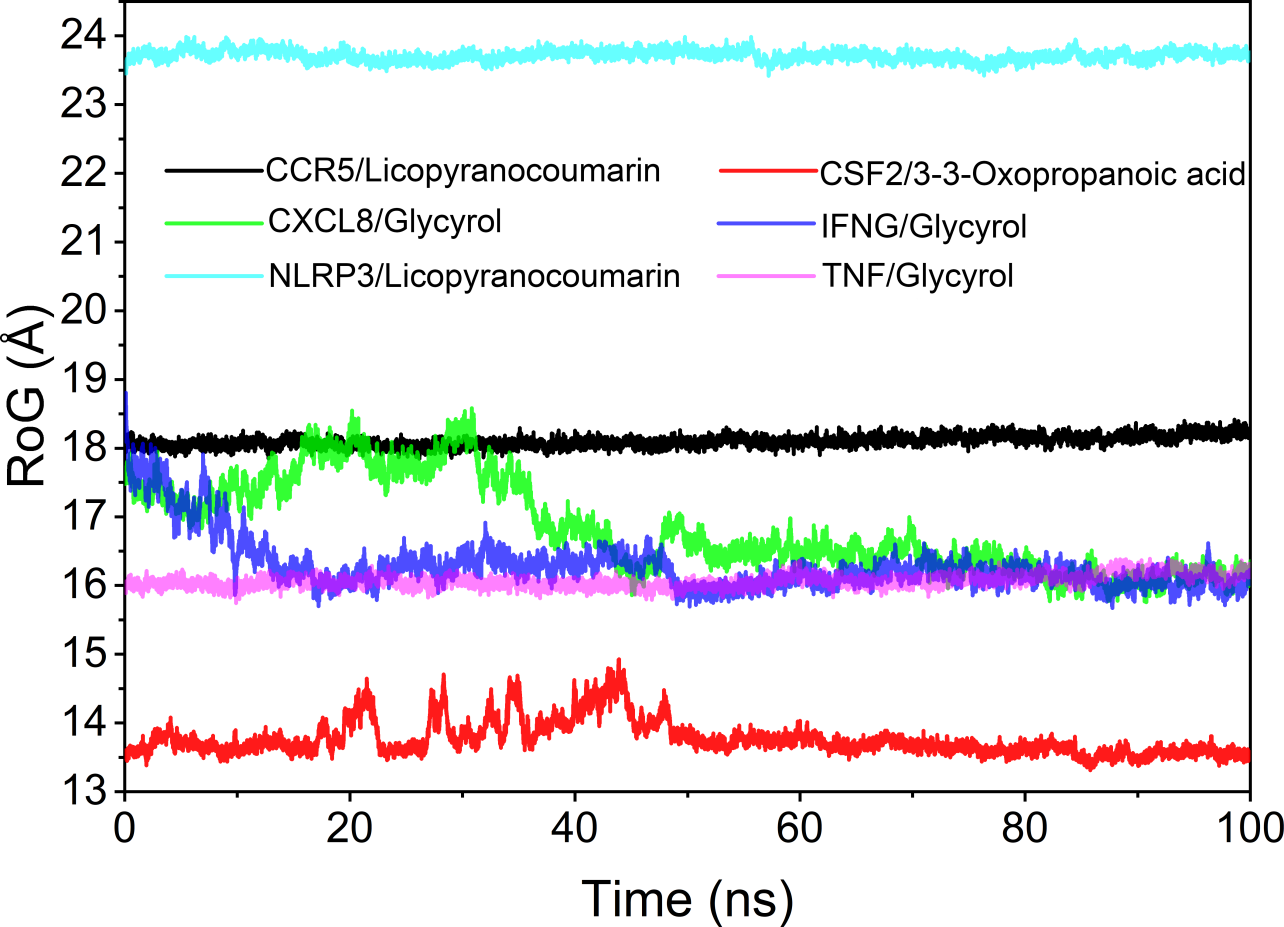
Analysis of radius of rotation (Rog).

The radius of rotation (Rog) can be used to reflect the tightness of the bonding of the complex system. The fluctuation size can very intuitively reflect the denseness of the composite or the system convergence. The values of Rog were TNF/Glycyrol, CCR5/Licopyranocoumarin, NLRP3/Licopyranocoumarin, CXCL8/Glycyrol, IFNG/Glycyrol and CSF2/3-3-Oxopropanoic acid from the largest to the smallest. The results of Rog were similar to the RMSD results, and the results indicated that TNF/Glycyrol, CCR5/Licopyranocoumarin and NLRP3/Licopyranocoumarin could maintain very stable binding state (**Figure 9**).

### Analysis of solvent accessible surface area

**Figure 10:**
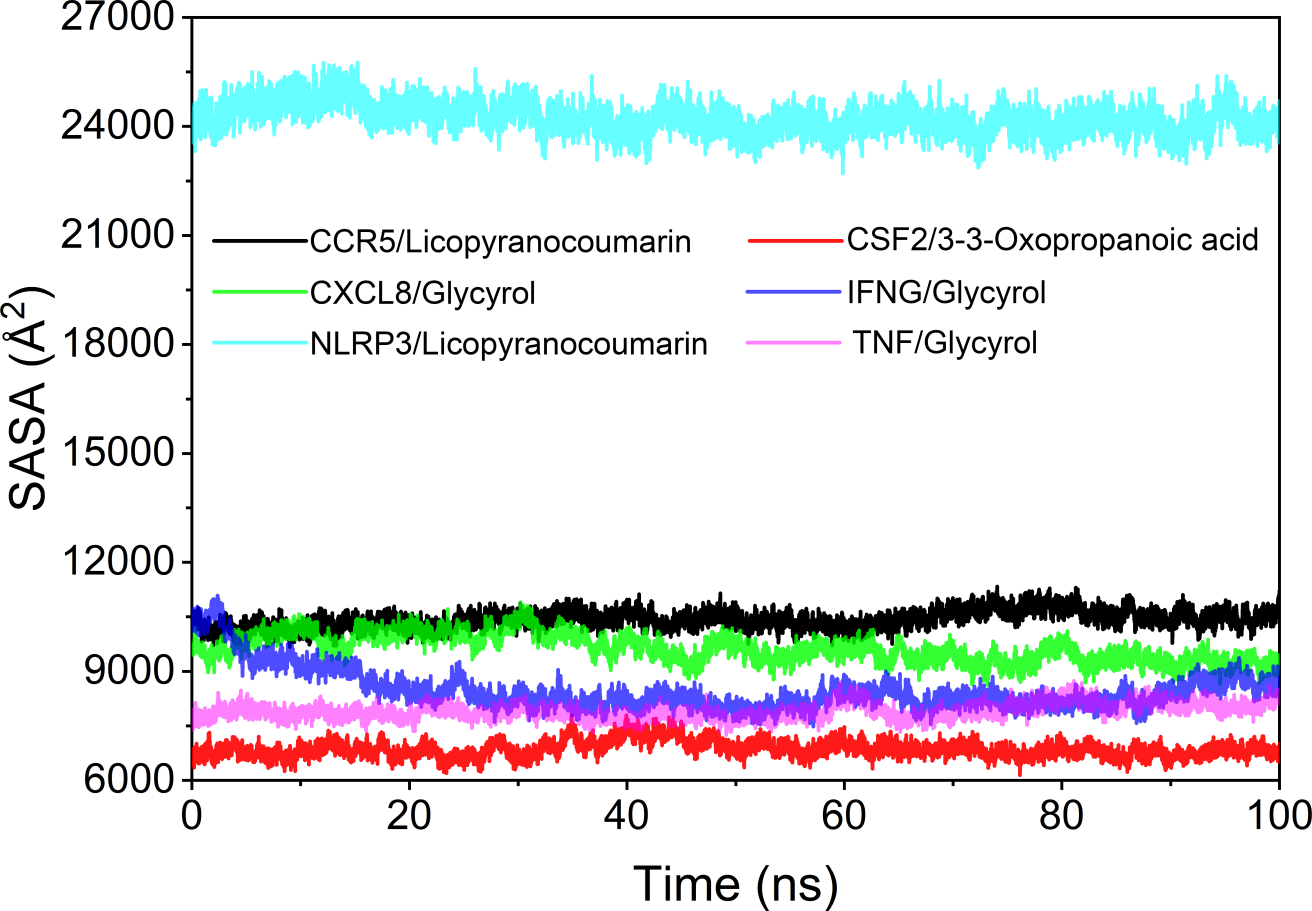
Analysis of solvent accessible surface area (SASA).

The solvent accessible surface area (SASA) is used to calculate the interface at which the complex is surrounded by the solvent. Because solvents behave differently under different conditions, SASA is a useful parameter for studying protein conformational dynamics in solvent environments. Based on the results of SASA fluctuation analysis, we could find that the fluctuation of CSF2/3-3-Oxopropanoic acid and NLRP3/Licopyranocoumarin were larger, which indicated that the exposure and burial area of the surface of this two proteins occur more changes (**Figure 10**).

## Discussion

We explored the mechanism of *Lianhua Qingwen granules* (LHQW) treats COVID-19 by computer simulations. This study found that active ingredients in *Lianhua Qingwen granules* (LHQW) may reduce cell damage and tissue destruction composed of COVID-19 by inhibiting the inflammatory response and regulating cell survival. Firstly, Glycyrol may inhibit activation of the inflammatory response and chemotaxis of inflammatory cells through IFNG and CXCL8 thereby reducing the inflammatory response. Glycyrol may also regulate TNF to reduce cell damage and death thereby alleviating damage from the inflammatory response caused by infection. Secondly, Licopyranocoumarin may impede release of inflammatory factors and spread of inflammatory response through CCR5 and NLRP3 thereby controlling the extent of inflammatory effects and the degree of tissue damage. Finally, 3-3-Oxopropanoic acid may regulate the differentiation of inflammatory cells and cell survival through CSF2 thereby reducing apoptosis.

Therefore, these results demonstrate that active ingredients in *Lianhua Qingwen granules* (LHQW) treats COVID-19 by regulating cell survival and inhibiting inflammatory response from multiple targets.

### Analysis of molecular docking and molecular dynamics

Molecular docking could be used to revealed strong affinity of active ingredients in *Lianhua Qingwen granules* (LHQW) (such as: Glycyrol Licopyranocoumarin and 3-3-Oxopropanoic acid) to protein targets (such as: IFNG, CXCL8, TNF, CCR5, NLRP3 and CSF2). And molecular dynamics results suggested that active ingredients in *Lianhua Qingwen granules* (LHQW) and protein could maintain a very stable binding state and thus exert effects in the treatment of COVID-19.

The binding of IFNG/Glycyrol indicated that the Glycyrol hydrogen bonds with Ala-8 and Leu-28 on IFNG, hydrophobic interaction with Leu-11, Phe-15, Phe-57, Leu-57 and Leu-30. In the CXCL8/Glycyrol, Glycyrol interacted with Glu-29 and Val-27 on CXCL8 protein by hydrogen bonding and with Val-58, Ile-61, Leu-25 and Val-27 by hydrophobic interactions. The binding of TNF/Glycyrol was mainly through hydrophobic interaction, Glycyrol interacted with Phe-144, Ala-145, Asp-143, Gln-67 and Tyr-141 on the TNF protein by hydrophobic interactions and with Glu-23 by hydrogen bonding. The binding free energies of CXCL8/Glycyrol, IFNG/Glycyrol, TNF/Glycyrol were -39.15 ± 1.96 kcal/mol, -34.16 ± 4.59 kcal/mol and -16.84 ± 1.92 kcal/mol. The number of CXCL8/Glycyrol, IFNG/Glycyrol and TNF/Glycyrol hydrogen bonds remained around 2 in the late simulation period.

Licopyranocoumarin bound to CCR5 and NLRP3 could form very stable complex. Licopyranocoumarin interacted with Arg-184, Tyr-232, Gln-41, Gly-44, Gly-46 and Ser-245 on CCR5 by hydrogen bonding and with Val-230 and Pro-149 by hydrophobic interactions. Licopyranocoumarin could form hydrogen bonding with Ser-626 and Arg-578 on NLRP3 protein, and also with Val-353, Pro-352, Tyr-632, Ile-411, Phe-575 and Thr-439 formed a hydrophobic interaction. The binding free energy of CCR5/Licopyranocoumarin and NLRP3/Licopyranocoumarin were -25.52 ± 2.86 kcal/mol and -38.17 ± 1.51 kcal/mol. The RMSD values of CCR5/Licopyranocoumarin and NLRP3/Licopyranocoumarin were small (values below 3.5 Å) and fluctuated very steadily.

The binding of the CSF2/3-3-Oxopropanoic acid was maintained mainly by hydrophobic interactions. The binding of the CSF2/3-3-Oxopropanoic acid was maintained by hydrophobic interactions. The small molecule 3-3-Oxopropanoic acid interacted with His-15 on the CSF2 protein by hydrogen bonding and with Leu-55, Leu-59, Phe-47, Ile-117 and Ile-19 by hydrophobic interactions. The binding free energies of CSF2/3-3-Oxopropanoic acid was -6.98 ± 5.02 kcal/mol. And the overall RMSF of CSF2/3-3-Oxopropanoic acid was less than 2.5 Å.

### Glycyrol may inhibit activation of the inflammatory response and chemotaxis of inflammatory cells through IFNG and CXCL8

Interferon-gamma (IFNG) is essential for immune and tumor control of intracellular pathogens. However, abnormal IFNG expression is associated with many auto-inflammatory and autoimmune diseases. GO enrichment analysis includes interferon-γ receptor binding and cytokine activity. KEGG enrichment analysis includes IL27-mediated signaling pathways. C-X-C Motif Chemokine Ligand 8 (CXCL8) is a major mediator of the inflammatory response, CXCL8 clears pathogens and protects the host from infection by attracting neutrophils and T cells. GO enrichment includes interleukin 8 receptor binding and chemokine activity. KEGG enrichment analysis includes GPCR downstream signaling and bacterial infection in the airway.

IFNG is selectively produced by immune cells (such as: T lymphocytes and NKT cells). And the receptor for IFNG (IFNGR) is present in almost all cell types. IFNG can induce antiviral, antiproliferative and immunomodulatory effects in the direction of inflammation (Schoenborn and Wilson, 2007; Nöst et al., 2019; Ozger et al., 2021). IFNG encodes soluble cytokines secreted by the innate and adaptive immune systems. Elevated IFNG levels have been found in COVID-19 patients, and elevated IFNG levels are thought to exacerbate cytokine storms (Xu et al., 2022). IFNG is mainly produced by CD4 T cells. IFNG secretion enhances macrophage microbicidal mechanisms and regulates Th17 cell and tissue damage (Petrone et al., 2021). CD4+ T cells have two inflammatory subpopulations, Th1 and Th17 cells. Th1 and Th17 perform their effector functions by producing inflammatory cytokines (including IFNG and IL-17). Melgaço et al. showed that stinging viral proteins lead to activation of CD4 T helper cell and CD8 T to produce IFNG (Melgaço et al., 2021). Therefore, inhibition of IFNG or IL-17 seems to improve clinical status of critically ill patients. Shahbazi et al. showed an overall reduction in pro-inflammatory Th1 and Th17 lymphocytes in critically ill patients with COVID-19. The reduced frequency of these lymphocytes may be due to their migration to the lungs of critically ill patients and this increases lung inflammation (Shahbazi et al., 2021; Mele et al., 2021). Some studies have shown that the active ingredients of LHQW (such as: lonicerae and glycyrrhizin) can exert inhibitory effects on IFNG and reduce IFNG expression (Nöst et al., 2019; Liu P et al., 2021).

CXCL8 can be rapidly induced by pro-inflammatory cytokines (such as: TNF and IL-1b) (Liu et al., 2016). Kaiser et al. showed that neutrophils were recruited to express CXCL8 in the lung, which in turn activates and enhances CXCL8 release from peripheral neutrophils (Ha et al., 2017). Kaiser et al. found high concentrations of CXCL8 in bronchoalveolar lavage (BAL) fluid from patients with COVID-19 (Kaiser et al., 2021). Studies have shown that LHQW can reduce the gene level of interleukin 8 (CXCL8) (Dong et al., 2014).

Therefore, Glycyrol may inhibit activation of the inflammatory response and chemotaxis of inflammatory cells through IFNG and CXCL8 thereby reducing the inflammatory response.

### Glycyrol may regulate TNF to reduce cell damage and death

Tumor necrosis factor alpha is a member of the TNF/TNFR cytokine superfamily (Balkwill, 2006). TNF-α can be released from the cell membrane by extracellular protein hydrolysis cleavage and functions as a cytokine. GO enrichment analysis includes cytokine activity and protein binding. KEGG enrichment analysis includes the regulation of dendritic cell developmental lineage pathways and cell survival.

TNF-α is an inflammatory cytokine produced by macrophages/monocytes during acute inflammation, and TNF-α is responsible for various intracellular signaling events (mediating the gene expression of growth factors, cytokines and transcription factors). TNF-α is also important in the fight against infection and cancer (Idriss and Naismith, 2000; Ramasamy and Subbian, 2021). And TNF-α affects different aspects of the immune system and regulates various pathological and physiological processes (Choudhary et al., 2021). TNF-α is also a major activator of IL-6 expression (Pons et al., 2020). Study found that TNF-α was a prominent feature of patient deterioration (Fara et al., 2020). The severity of COVID-19 is associated with damage produced by cytokine storm and type I IFN (IFN-α and IFN-β) (Ramasamy and Subbian, 2021; Krämer et al., 2021). Interestingly, the numbers of total T cells, CD4 T and CD8 T cells were negatively correlated with TNF-α, IL-6 levels, respectively. This leads to the conclusion that T cells are reduced and depleted in COVID-19 patients. Cytokines (such as IL-6 and TNF-α) may be involved in T-cell reduction (Diao et al., 2020). Study has been shown that most patients suffer from lymphocytopenia with elevated serum pro-inflammatory cytokine levels (such as: TNF-α, IL-6) (Khanmohammadi and Rezaei, 2021). In addition, Norooznezhad et al. found that NF-κB, a very critical transcription factor in inflammation (especially cytokine storm) may lead to the expression of IL-6 and TNF-α (Pons et al., 2020; Norooznezhad and Mansouri, 2021; Attiq et al., 2021). Study has been shown that LHQW can inhibit virus-induced NF-κB activation and alleviate virus-induced gene expression of IL-6, TNF-α (Shen and Yin, 2021).

Therefore, Glycyrol may also regulate TNF to reduce cell damage and death thereby alleviating damage from the inflammatory response caused by infection.

### Licopyranocoumarin may impede the release of inflammatory factors and the spread of the inflammatory response through CCR5 and NLRP3

C Motif Chemokine Receptor 5 (CCR5, also known as CD195) is a G protein-coupled receptor (GPCR) (Zeng et al., 2022). CCR5 is expressed on the surface of leukocytes, especially T-CD4^+^ cells. CCR5 mediates the migration of macrophages to inflammatory areas facilitating the release of inflammatory cytokines and the amplification of immune responses (Iannaccone et al., 2020). GO enrichment analysis includes G protein-coupled receptor activity. KEGG enrichment analysis includes selective expression of chemokine receptors and autophagic pathways during T cell polarization. Activation of NLRP3 as a key component of the innate immune system plays a critical role in host defense against bacteria, fungi and viruses, and NLRP3 is also associated with metabolic and inflammatory conditions. GO enrichment includes peptidoglycan binding. KEGG enrichment analysis includes activation of NF-KappaB by PKR and inflammatory vesicles.

CCR5 may play a role in the inflammatory response to coronavirus infection. Study has been suggested that the CCR5 pathway is a suppressor of immune hyperactivation in severe COVID-19 (Cuesta-Llavona et al., 2021; Dieter et al., 2022). Lungs infected with SARS-COV-2 show upregulation of chemokines (including CCL4, CCL8, and CCL11), and all of these factors share CCR5 as their receptor (Hachim et al., 2020; Zeng et al., 2022). The active ingredient of LHQW can inhibit the secretion of CCL5 thereby inhibiting the interaction between CCL5 and CCR5, and suppressing the inflammatory response (Ko et al., 2006).

NLR Family Pyrin Domain Containing 3 (NLRP3) inflammatory vesicles are one of the most important components of the innate immune system and NLRP3 significantly enhances inflammation by increasing the production of IL-18 and gasdermin D (GSDMD) (López-Reyes et al., 2020; Freeman and Swartz, 2020; Saeedi-Boroujeni et al., 2021). The immune overreaction and cytokine storm in SARS-CoV-2 infection may be associated with NLRP3 inflammatory vesicle activation. In SARS-CoV-2 infection, NLRP3 inflammatory vesicle activation leads to the stimulation and synthesis of natural killer cells (NKs), NF-κB and interferon γ (INF-γ) (Batiha et al., 2021; Rodrigues et al., 2021). The active ingredient of LHQW inhibits NLRP3 inflammatory vesicles (Liu et al., 2019), and LHQW blocks the onset of apoptosis and inflammatory response by inhibiting the activation of NLRP3 (Chao et al., 2022).

Therefore, Licopyranocoumarin may impede release of inflammatory factors and spread of inflammatory response through CCR5 and NLRP3 thereby controlling the extent of inflammatory effects and the degree of tissue damage.

### 3-3-Oxopropanoic acid may regulate the differentiation of inflammatory cells and cell survival through CSF2

Granulocyte macrophage colony-stimulating factor 2 (CSF2) is a key cytokine. CSF2 affects the survival, proliferation and differentiation of dendritic cells and macrophages by stimulating myeloid cells (Sielska et al., 2020; Saita et al., 2022). GO enrichment analysis includes inflammatory factor activity. KEGG enrichment analysis includes selective expression of chemokine receptors during T cell polarization and dendritic cell developmental lineage pathways.

Study has been shown that blockade of CSF2 signaling reduces airway inflammation and hyperresponsiveness in mouse models of environmentally induced lung injury and asthma (Burkhardt et al., 2012; Gilchrist et al., 2021). Lung tissue-resident memory-like TH17 cells are predominant immune cell type in bronchoalveolar lavage fluid (BAL) expressing the cytokine GM-CSF. GM-CSF were significantly elevated in the serum of patients with severe COVID-19 compared to patients with normal COVID19. Study has been shown that an enhanced frequency of GM-CSF/IFNG replicating T cells was found in the blood of COVID-19 patients and appeared to correlate with disease activity. Zhao et al. suggested that CSF2/GM-CSF expressing cells were present in the lung and co-express IL17A. Lung T+RM17 cells are a potential coordinating factor for excessive inflammation in severe COVID-19 (Schett et al., 2020; Zhao et al., 2021). Li et al. identified enhanced Th17 cell differentiation and cytokine responses in COVID-19 (Li et al., 2021). Some studies have analyzed and identified genes specifically expressed by SARS-CoV-2 infection, as well as genes altered due to coronavirus-2 and/or other respiratory viral infections. In particular, CSF2 expression appears to be associated with neo-coronavirus disease (Chandrashekar et al., 2021; Ferrarini et al., 2021). Cheng et al. determined that the cytokine gene CSF2 was upregulated after SARS-CoV-2 infection (Cheng et al., 2021). SARS-CoV-2 infection can activate T cells. Activated T cells rapidly proliferate and secrete granulocyte-macrophage colony-stimulating factor (GM-CSF) and interleukin-16 (IL-16) (Shen and Yin, 2021).

Therefore, 3-3-Oxopropanoic acid may regulate the differentiation of inflammatory cells and cell survival through CSF2 thereby reducing apoptosis.

## Conclusion

We explored the mechanism of *Lianhua Qingwen granules* (LHQW) treats COVID-19 by molecular docking and molecular dynamics. We found that the active ingredients in LHQW not only reduce cell damage and tissue destruction by inhibiting the inflammatory response through CSF2, CXCL8, CCR5 and IFNG, but also regulate cell survival and growth through NLRP3 and TNF.

Therefore, the active ingredients in *Lianhua Qingwen granules* (LHQW) treats COVID-19 by regulating cell survival and inhibiting inflammatory response from multiple targets.

## Data availability statement

The datasets presented in this study can be found in online repositories. The names of the repository/repositories and accession number(s) can be found in the article/supplementary material.

## Author contributions

Author contributions: J-FC, YG, MW, LX, XZ (12^th^ author) contributed to the conception of the study. J-FC, MW, SC, HH, XZ (7^th^ author), Y-CP, X-FS, JQ contributed significantly to analysis and manuscript preparation. J-FC, YG, MW, LX performed the data analyses and wrote the manuscript. XZ (12^th^ author), J-FC, Y-LW. All authors contributed to the article and approved the submitted version.

## Funding

This study was supported by “High-level talent research start-up fund of The First Affiliated Hospital of Chengdu Medical College (CYFY-GQ27)”.

## Conflict of interest

The authors declare that the research was conducted in the absence of any commercial or financial relationships that could be construed as a potential conflict of interest.

## Publisher’s note

All claims expressed in this article are solely those of the authors and do not necessarily represent those of their affiliated organizations, or those of the publisher, the editors and the reviewers. Any product that may be evaluated in this article, or claim that may be made by its manufacturer, is not guaranteed or endorsed by the publisher.
